# Examination of the Impact of CYP3A4/5 on Drug–Drug Interaction between Schizandrol A/Schizandrol B and Tacrolimus (FK-506): A Physiologically Based Pharmacokinetic Modeling Approach

**DOI:** 10.3390/ijms23094485

**Published:** 2022-04-19

**Authors:** Qingfeng He, Fengjiao Bu, Qizhen Wang, Min Li, Jiaying Lin, Zhijia Tang, Wen Yao Mak, Xiaomei Zhuang, Xiao Zhu, Hai-Shu Lin, Xiaoqiang Xiang

**Affiliations:** 1 Department of Clinical Pharmacy and Pharmacy Administration, School of Pharmacy, Fudan University, Shanghai 201203, China; qf_he@fudan.edu.cn (Q.H.); fbu13@fudan.edu.cn (F.B.); wangqz16@fudan.edu.cn (Q.W.); 19211030067@fudan.edu.cn (M.L.); 21211030054@fudan.edu.cn (J.L.); zjtang@fudan.edu.cn (Z.T.); makwenyao@gmail.com (W.Y.M.); xiaozhu@fudan.edu.cn (X.Z.); 2 Clinical Research Centre, Hospital Pulau Pinang, Pinang 10450, Malaysia; 3 Institute for Clinical Research, National Institute of Health, Shah Alam 40170, Malaysia; 4 State Key Laboratory of Toxicology and Medical Countermeasures, Beijing Institute of Pharmacology and Toxicology, Beijing 100850, China; xiaomeizhuang@163.com; 5 College of Pharmacy, Shenzhen Technology University, Shenzhen 518118, China

**Keywords:** physiologically based pharmacokinetic (PBPK), Wuzhi capsule (WZC), tacrolimus (FK-506), CYP3A5 polymorphism, drug–drug interaction (DDI), schizandrol A (SZA), schizandrol B (SZB)

## Abstract

Schizandrol A (SZA) and schizandrol B (SZB) are two active ingredients of Wuzhi capsule (WZC), a Chinese proprietary medicine commonly prescribed to alleviate tacrolimus (FK-506)-induced hepatoxicity in China. Due to their inhibitory effects on cytochrome P450 (CYP) 3A enzymes, SZA/SZB may display drug–drug interaction (DDI) with tacrolimus. To identify the extent of this DDI, the enzymes’ inhibitory profiles, including a 50% inhibitory concentration (IC_50_) shift, reversible inhibition (RI) and time-dependent inhibition (TDI) were examined with pooled human-liver microsomes (HLMs) and CYP3A5-genotyped HLMs. Subsequently, the acquired parameters were integrated into a physiologically based pharmacokinetic (PBPK) model to quantify the interactions between the SZA/SZB and the tacrolimus. The metabolic studies indicated that the SZB displayed both RI and TDI on CYP3A4 and CYP3A5, while the SZA only exhibited TDI on CYP3A4 to a limited extent. Moreover, our PBPK model predicted that multiple doses of SZB would increase tacrolimus exposure by 26% and 57% in CYP3A5 expressers and non-expressers, respectively. Clearly, PBPK modeling has emerged as a powerful approach to examine herb-involved DDI, and special attention should be paid to the combined use of WZC and tacrolimus in clinical practice.

## 1. Introduction

Pharmacokinetic drug interaction can occur when one drug affects the absorption, distribution, metabolism, or excretion (ADME) of another drug [1]. The complexity of ADME processes is attributed to numerous functional proteins with individual variations. Since many therapeutic agents undergo metabolism by cytochrome P450 (CYP450) enzymes, great attention has been given to drug–drug interaction (DDI) mediated by CYP450 enzyme inhibition [2,3]. Traditional Chinese medicine (TCM) has increasingly gained in popularity in Western countries, especially during the COVID-19 pandemic. Some clinical reports have suggested that the coadministration of conventional (Western) medicine with TCM could reduce toxicity and/or enhance therapeutic efficacy [4,5]. However, TCM products could cause DDI and affect the metabolism and clearance of various drugs via CYP450 inhibition [6]. The composition of TCM preparations is complicated by the uncertain contents of bioactive ingredients, which fluctuate wildly due to the varied growing origins and culturing techniques. The lack of consistency and standardization makes it difficult to evaluate the DDIs between TCM and Western medicine in clinical settings. As conventional medicine is commonly used together with TCM in China, there is an urgent need to appraise the safety and effectiveness of this integrative practice.

**Figure 1:**
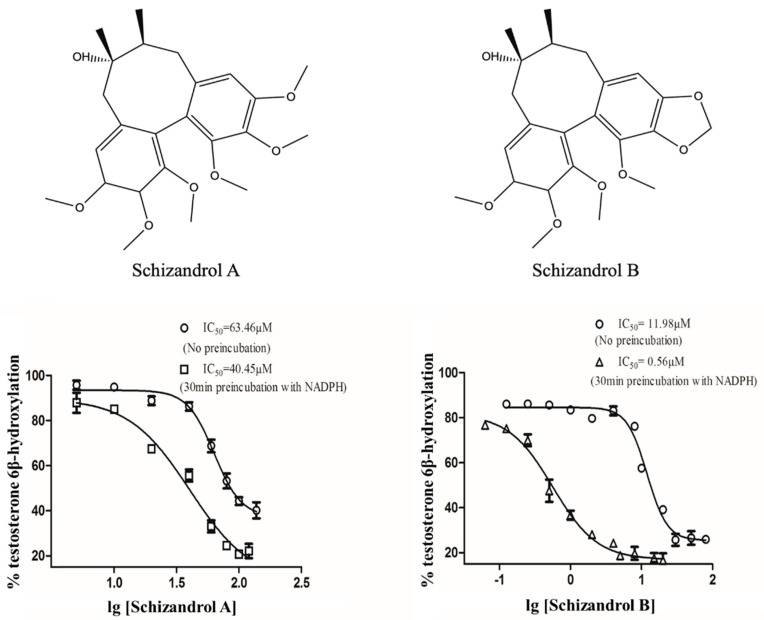
IC_50_ values of SZA and SZB in pooled HLMs with and without 30-minute preincubation time. Various concentrations of SZA (0–100 μM) and SZB (0–20 μM) were used. The experiments were conducted in triplicate.

Wuzhi capsule (WZC) is an ethanol extract of the *Schisandra sphenanthera* with major bioactive chemical components including schisantherin A (STA), schisandrin A (SIA), schizandrol A (SZA) and schizandrol B (SZB). Since WZC alleviates drug-induced liver toxicity and damage, it is commonly co-prescribed with hepatotoxic drugs in China [7]. Tacrolimus (FK-506), a first-line immunosuppressive agent indicated for solid-organ transplantation, is mainly metabolized by CYP3A4 and CYP3A5 and eliminated in the liver [8,9,10]. Due to its narrow therapeutic index and pharmacokinetic variations, patients with different pathophysiological conditions are prone to suffer from overexposure or underexposure [11]. The coadministration of CYP3A inhibitor can increase tacrolimus’s blood concentration, resulting in nephrotoxicity and a higher infection risk. Although commonly prescribed together with tacrolimus, WZC significantly increased the whole blood concentration of tacrolimus [12,13]. Mechanistic studies further revealed the inhibitory potency of STA and SIA, active constituents of *Schisandra* *sphenanthera*, on CYP3A4 and CYP3A5 [1,14,15,16,17]. Similarly, SZA and SZB (structures displayed in Figure 1) also affected CYP3A enzymes, and the systemic exposure of tacrolimus was increased by 598.4% in rats after the oral administration of SZB [16].

The pharmacogenetic polymorphism of CYP3A5 also has a significant impact on the metabolism of tacrolimus [18,19,20]. A decreased trough concentration had been observed in CYP3A5 expressers of CYP3A5* 1 allele (active genotype) in comparison to CYP3A5* 3 alleles (inactive genotype) [21]. Therefore, dose adjustment is recommended by the Clinical Pharmacogenetics Implementation Consortium (CPIC), while CYP3A5* 1 allele carriers should receive 1.5 to 2 times higher doses of tacrolimus to achieve blood exposure similar to CYP3A5 non-expressers [18].

Physiologically based pharmacokinetic (PBPK) modeling has been used to predict concentration-time profiles and, therefore, adapted in DDI investigations [22,23,24,25]. When dealing with herbal medicines/natural products, PBPK modeling applies in vitro to in vivo extrapolation and predicts pharmacokinetic drug interactions and toxicological profiles in a simulation manner [22,26]. We successfully quantified the contribution of STA and SIA to the DDI between tacrolimus and WZC in previous studies [14,17]. However, the impact of the pharmacogenetic polymorphism of CYP3A5 on the interaction between tacrolimus and SZA/SZB remains largely unclear. In this study, we first assessed the inhibitory potencies of SZA and SZB on CYP3A4/5 and then examined the contribution of CYP3A5 polymorphism to the variability of the interaction between tacrolimus and WZC by constructing a PBPK–DDI model integrated with pharmacogenomic factor as a quantitative parameter. Hopefully, the information obtained in this study will improve our understanding of the DDI between WZC and tacrolimus and enhance their clinical safety and effectiveness.

## 2. Results

### 2.1. IC_50_ Shift

The IC_50_ values of SZA (63.46 μM for no preincubation, 40.45 μM for preincubation with NADPH) and SZB (11.98 M for no preincubation, 0.56 μM for preincubation with NADPH) are shown in Figure 1. The IC_50_ shift values were calculated to be 1.57 for SZA and 21.39 for SZB.

### 2.2. RI Assay

**Figure 2:**
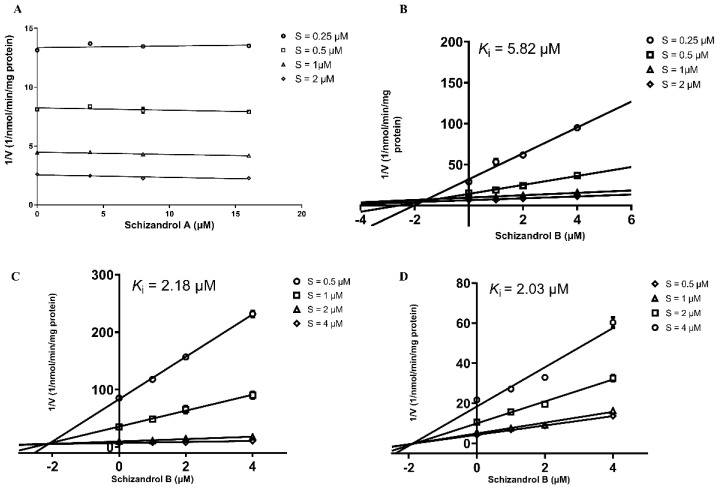
Dixon plots of SZA (**A**) and SZB (**B**) on tacrolimus metabolism mediated by CYP3A metabolism in pooled HLMs. Various concentrations of tacrolimus (0.25, 0.5, 1, 2 μM), SZA (0, 4, 8, 16 μM) and SZB (0, 1, 2, 4 μM) were used. Dixon plot of SZB on CYP3A4 (**C**) and CYP3A5 (**D**) in genotyped HLMs. Various concentrations of tacrolimus (0.5, 1, 2, 4 μM) and SZB (0, 1, 2, 4 μM) were used. Experiments were conducted in triplicates.

Dixon plots were used to yield the K_i_ values for the inhibition by SZA or SZB. The reaction rate did not change significantly as the SZA’s concentration increased in the incubation with the pooled HLMs (Figure 2A), demonstrating little reversible inhibition on CYP3A by SZA. However, as shown in Figure 2B, the SZB presented a competitive and reversible inhibition pattern with a K_i_ value of 5.82 μM. A further evaluation of the inhibitory pattern of SZB on CYP3A4 and CYP3A5 with different genotyped HLMs was performed, and the values of K_i_ (2.18 and 2.03 μM, respectively) are displayed in Figure 2C,D.

### 2.3. TDI Assay

**Figure 3:**
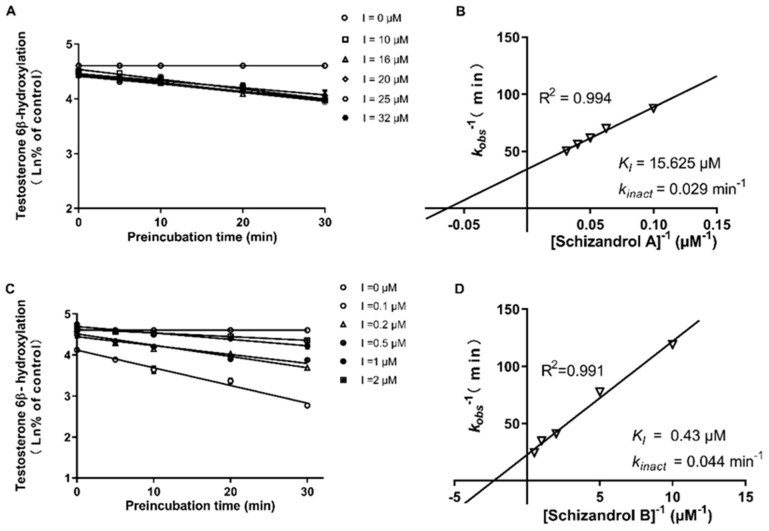
Dixon plots of SZA (**A**,**B**) and SZB (**C**,**D**) on CYP3A inactivation in pooled HLMs. Various concentrations of SZA (0, 10, 16, 20, 25, 32 μM) and SZB (0, 0.1, 0.2, 0.5, 1, 2 μM) were pre-incubated at 37 °C for 0, 5, 10, 20 and 30 min in 0.1 M PBS. (**A**,**C**) were plotted with the log of percentages of control activity versus preincubation time. (**B**,**D**) were plotted with the half-life of enzyme inactivation versus the inverse of the SZA or SZB concentration. Each point represents the mean of triplicate experiments.

**Figure 4:**
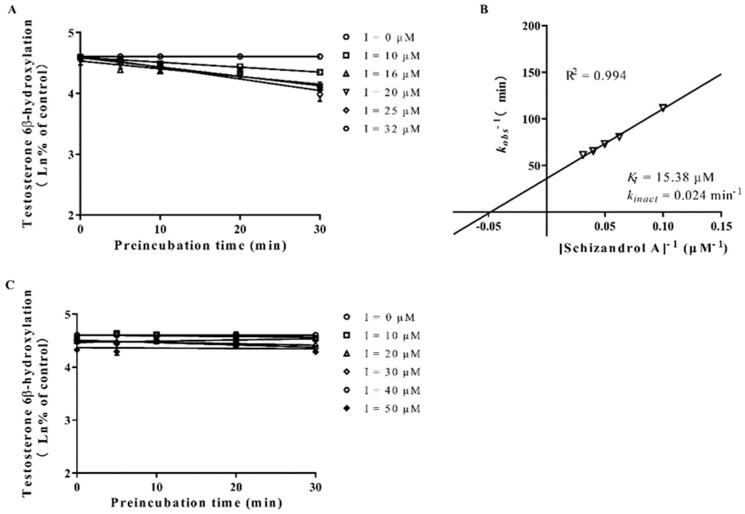
Dixon plots of SZA on CYP3A4 (**A**,**B**) and CYP3A5 (**C**) inactivation in genotyped HLMs. Various concentrations of SZA (0, 10, 16, 20, 25, 32 μM for CYP3A4 and 0, 10, 20, 30, 40, 50 μM for CYP3A5) were pre-incubated at 3 °C for 0, 5, 10, 20 and 30 min in 0.1 M PBS. (**A**,**C**) were plotted with the log of the percentage of control activity versus preincubation time. (**B**) was plotted with the half-life of enzyme inactivation versus the inverse of the SZA concentration. Each point represents the mean of triplicate experiments.

**Figure 5:**
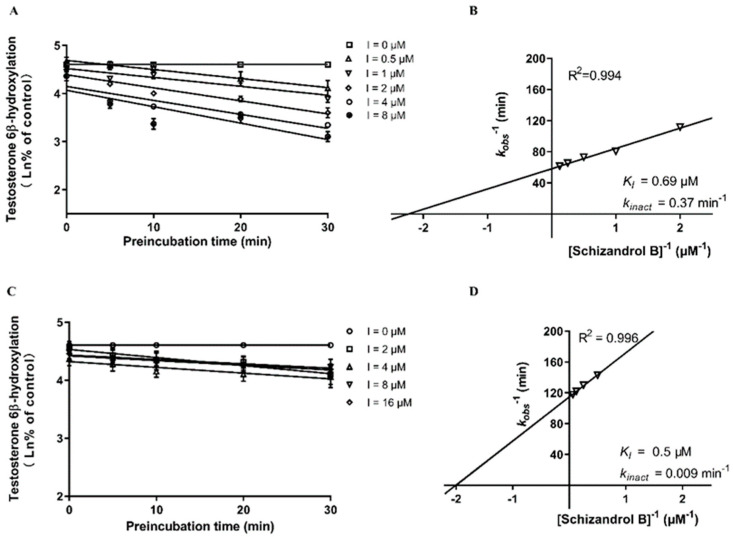
Dixon plots of SZB on CYP3A4 (**A**,**B**) and CYP3A5 (**C**,**D**) inactivation in genotyped HLMs. Various concentrations of SZB (0, 0.5, 1, 2, 4, 8 μM for CYP3A4 and 0, 2, 4, 8, 16 μM for CYP3A5) were pre-incubated at 37 °C for 0, 5, 10, 20 and 30 min in 0.1 M PBS. (**A**,**C**) were plotted with the log of the percentage of control activity versus preincubation time. (**B**,**D**) were plotted with the half-life of enzyme inactivation versus the inverse of the SZB concentration. Each point represents the mean of triplicate experiments.

The formation of 6β-hydroxytestosterone was used to measure the enzymatic activity of CYP3A4 and 3A5. As presented in Figure 3, both the SZA and the SZB exhibited a powerful TDI on the CYP3A. The k_inact_ and K_I_ values of the SZA on the CYP3A were 0.029 min^−^^1^ and 15.625 μM, respectively, compared with 0.044 min^−^^1^ and 0.43 μM for SZB. Further investigation on the CYP3A4 and CYP3A5 separately indicated that the SZA had a slight TDI on the CYP3A4 (k_inact_ = 0.024 min^−^^1^, K_I_ = 15.38 μM) but not on the CYP3A5 (Figure 4). The SZB demonstrated a strong irreversible inhibition on both the CYP3A4 (k_inact_ = 0.37 min^−^^1^, K_I_ = 0.69 μM) and the CYP3A5 (k_inact_ = 0.009 min^−^^1^, K_I_ = 0.5 μM) (Figure 5).

### 2.4. Model Establishment and Validation

#### 2.4.1. PBPK Models for SZA and SZB

The observed data were retrieved from the published literature to predict the plasma concentrations of the SZA and the SZB after the oral administration of WZC in healthy Chinese populations [27]. Even though the amounts of the SZA and SZB in the WZC were small, the in vivo exposure was relatively large. Due to the difficulty of achieving the reported area under the curve (AUC) with the actual proportion of SZA and SZB, we used a virtual dose calculated by Simcyp^®^ and established PBPK models for single doses of SZA (33.6 mg) and SZB (10.8 mg) in healthy volunteers. As shown in Equation (1), the fold error was calculated to measure the accuracy of the simulation, with values of less than 2 indicating a precise prediction.

(1) $$\mathrm{Fold}\;\mathrm{error}=\frac{\mathrm{predicted}\;\mathrm{value}}{\mathrm{observed}\;\mathrm{value}}\;\left(\mathrm{if}\;\mathrm{predicted}>\mathrm{observed}\right)\;\mathrm{Fold}\;\mathrm{error}\;=\frac{\mathrm{observed}\;\mathrm{value}}{\mathrm{predicted}\;\mathrm{value}}\;\left(\mathrm{if}\;\mathrm{observed}\;>\mathrm{predicted}\right)$$

**Figure 6:**
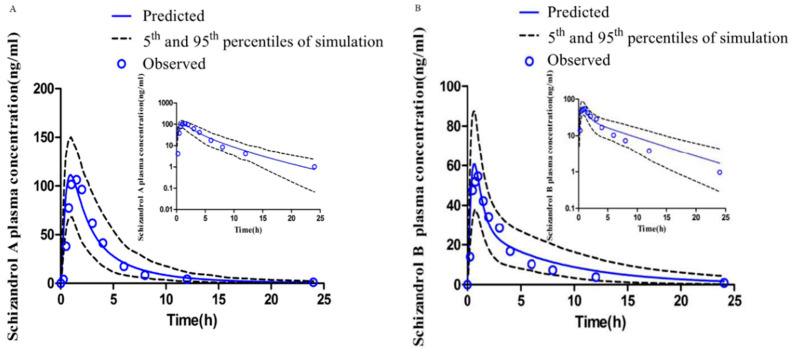
Simulation of plasma-concentration-time profiles of SZA (**A**) after a single oral dose of 33.6 mg and SZB (**B**) after a single oral dose of 10.8 mg in healthy Chinese patients generated by Simcyp^®^.

Comparing the predicted data with the observed data, we reported that all of the fold error values for the pharmacokinetic parameters in vivo were less than 2 (shown in Table 1), with good linearity (shown in Figure 6).

#### 2.4.2. DDI Simulation Modeling

Based on the developed human PBPK models of tacrolimus, SZA and SZB, we integrated the acquired RI kinetic parameter (K_i_), the TDI kinetic parameters (K_I_ and k_inact_) and the aforementioned kinetic parameters to form a DDI simulation model. Two sets of dosing protocols were created. In the first, the patients took a single dose of inhibitor (33.6 mg SZA or 10.8 mg SZB) with 2 mg tacrolimus; in the second, the patients received 16.8 mg of SZA or 5.4 mg of SZB twice daily for 13.5 days, followed by a single tacrolimus dose of 2 mg on day 14. Three different cases with different inhibition patterns were developed to distinguish the contributions of the RI and TDI to the increase in tacrolimus AUC in the CYP3 A5 expressers and non-expressers, respectively. Case #1 only includes the RI, #2 only covers the TDI and #3 combines both the RI and the TDI.

#### 2.4.3. DDI Prediction in CYP3A5 Expressers

Under the conditions of case #3, the predicted AUC of the tacrolimus after a single oral dose of SZB was increased by 15% to 72.12 ng/mL·h, while multiple doses of SZB increased the AUC of tacrolimus by 23% to 76.68 ng/mL·h. In the single-dose simulation, the RI (case #1) and TDI (case #2) by SZB increased the AUC of the acrolimus by 12% and 4%, respectively, while 13% increases were noticed in both multiple-dose settings. However, the coadministration of SZA had little influence on the blood concentrations of tacrolimus in both single and multiple-dose settings.

#### 2.4.4. DDI Prediction in CYP3A5 Non-Expressers

**Figure 7:**
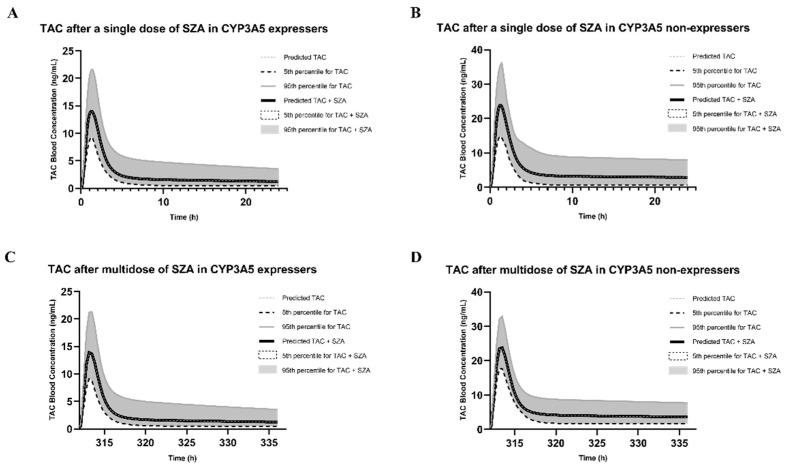
Change in tacrolimus exposure after a single dose of 33.6 mg SZA in CYP3A5 expressers (**A**) and CYP3A5 non-expressers (**B**); and after multiple doses of SZA (16.8 mg b.i.d. for 13.5 days) in CYP3A5 expressers (**C**) and CYP3A5 non-expressers (**D**).

**Figure 8:**
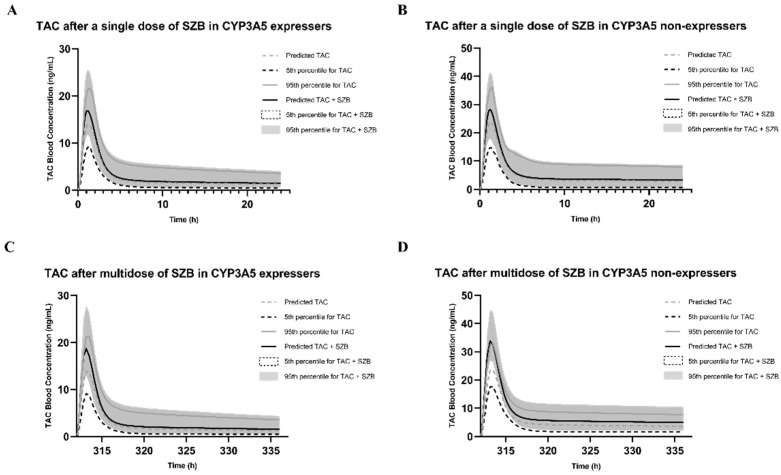
Change in tacrolimus exposure after a single dose of 10.8 mg SZB in CYP3A5 expressers (**A**) and CYP3A5 non-expressers (**B**); and after multiple doses of SZB (5.4 mg b.i.d. for 13.5 days) in CYP3A5 expressers (**C**) and CYP3A5 non-expressers (**D**).

Under the conditions of case #3, the predicted AUC of the tacrolimus in the blood after a single oral dose of SZB was increased by 14% to 135.01 ng/mL·h, while multiple doses of SZB increased the AUC of the tacrolimus by 57% to 185.61 ng/mL·h. The RI and TDI by SZB in the single-dose simulation increased the AUC of the tacrolimus by 9% and 6%, respectively, compared to the 26% and 47% increases in the multidose setting. In the simulation with the SZA, only a small alteration in the AUC ratio (AUCR) via the TDI, with values of 1.10 and 1.16, was obtained between the single-dose and multiple-dose scenarios, respectively. The results are presented in Figure 7 and Figure 8 and Table 2.

## 3. Discussion

The combined use of tacrolimus and WZC led to increased blood concentrations of tacrolimus, with AUC changes ranging from 57% to 366% [28]. Similarly, SZB led to a 200% increase in bosutinib exposure [29], indicating the urgent clinical need to identify potential interactions when co-prescribing WZC. As multiple active ingredients of WZC inhibit CYP3A4 and CYP3A5, it is challenging to elucidate the contributions of individual components. Furthermore, the polymorphism of the CYP3A5 enzyme causes tacrolimus metabolism to differ among patient populations; CYP3A5 expressers generally have decreased concentrations of tacrolimus compared to non-expressers. Our previous work demonstrated the inhibitory patterns of STA and SIA and integrated them into a PBPK–DDI model to predict the interaction with tacrolimus [14,17]. However, SZA and SZB are two further important components of WZC that possess the potential to cause DDI [30,31,32]. Therefore, we first used in vitro experimental approaches to investigate the inhibitory profiles of SZA and SZB on CYP3A enzymes. Subsequently, the PBPK model was developed and validated to predict the changes in tacrolimus exposure after the coadministration of SZA or SZB in CYP3A5 expressers or non-expressers. The IC_50_ shift values for both the SZA and the SZB were greater than 1.5, suggesting the potential existence of time-dependent inhibition [33]. The RI and TDI assays were conducted with pooled HLMs to identify the inhibitory patterns. Our results found that the SZA only inhibited the CYP3A in a time-dependent manner (K_I_ = 15.625 μM, k_inact_ = 0.029 min^−1^, k_inact_/K_I_ = 1.856 mL/min/μmol), while the SZB exhibited both reversible and irreversible inhibition (K_i_ = 5.82 μM, K_I_ = 0.43 μM, k_inact_ = 0.044 min^−1^, k_inact_/K_I_ = 102.33 mL/min/μmol). Clearly, the SZB displayed a superior inhibitory potency on CYP3A to the SZA. Subsequently, the metabolic study was carried out on the genotyped HLMs (CYP3A5 *1/*3 and CYP3A5 *3/*3) to examine the inhibitory effect of the SZA/SZB on the CYP3A4 and CYP3A5, respectively. The SZA only demonstrated an irreversible inhibitory pattern (K_I_ = 15.38 μM, k_inact_ = 0.024min^−1^, k_inact_/K_I_ = 1.56 mL/min/μmol) on the CYP3A4; however, the SZB exhibited both RI and TDI patterns on the CYP3A4 (K_i_ = 2.03 μM, K_I_ = 0.69 μM, k_inact_ = 0.37 min^−1^, k_inact_/K_I_ = 536.2 mL/min/μmol) and the CYP3A5 (K_i_ = 2.18 μM, K_I_ = 0.5 μM, k_inact_ = 0.009 min^−1^, k_inact_/K_I_ = 18 mL/min/μmol). Our results confirmed the findings of prior studies, according to which SZB caused moderate-to-strong CYP3A inhibition both reversibly and irreversibly, while SZA exhibited weak potency via TDI [31,34]. Furthermore, we identified that the SZB had a stronger inhibitory activity on the CYP3A4 than on the CYP3A5. A significant potency drop was noted in the assay with the pooled HLMs compared to the HLM with the CYP3A5 *3/*3 genotype. This finding could be attributed to the higher affinity and lower potency of SZB on CYP3A5 resulting in the dilution of the inhibitory effect. In this case, CYP3A5 expressers are expected to display higher variability in their tacrolimus metabolic/plasma profiles due to SZB inhibition.

Even though SZA and SZB only account for a small proportion of WZCs, their high blood levels were occasionally observed after oral administration [35]. This disparity could be explained by increased bioavailability or mutual biotransformation internally, caused by the presence of other ingredients in the compounded products [36]. Obvoiusly, it is challenging to perform an in vivo study to investigate the whole product, considering the unidentifiable pharmacokinetic and pharmacodynamic interactions among the active components. In addition to its cost-effectiveness, PBPK modeling is particularly helpful for integrating the pharmacokinetic properties of individual agents. Based on the actual blood concentrations, we set the virtual doses of the SZA and the SZB. At doses of 33.6 mg for SZA and 10.8 mg for SZB, our models display excellent fitness, with a fold error within 2.

Along with the established model for tacrolimus, we developed a PBPK–DDI model to investigate the impact of CYP3A5 genotypes on interactions with SZA or SZB. Interestingly, little influence was found on tacrolimus’ systemic exposure after a single dose of SZA regardless of CYP3A5 expression. In the CYP3A5 non-expressers, the SZA increased the AUC of the tacrolimus by 16% after multiple doses. These findings corroborated the results suggesting the weak TDI on the CYP3A4 mentioned above. On the other hand, single doses of SZB increased the AUC of the tacrolimus by 15% and 14% in the CYP3A5 expressers and non-expressers, respectively. This DDI was more potent in the multiple-dose settings.

It could be concluded that the SZB exhibited a more significant DDI in the CYP3A5 non-expressers, i.e., that the TDI on the CYP3A4 of the SZB contributed the most. Compared with our previous findings on STA and SIA [17], we noticed much weaker interactions between the SZA/SZB and the tacrolimus. It is worth mentioning that SZB contains a methylenedioxy moiety that is not present in SZA (Figure 1). A previous study suggested an association between this functional group and irreversible inhibition on CYP450 enzymes [37]; this could explain why the SZB displayed stronger inhibitory potency. However, further investigation is required to elucidate the structure–inhibitory-activity relationship.

The present study has some limitations. Firstly, transplantation patients were not included in our population database, causing potential bias in the simulation. Secondly, our models were restricted to examining the interaction between individual compounds and not the whole WZC; however, the latter is more clinically relevant. Such limitations could be overcome by further explorations and implementations of integrated PBPK models with different individual components. The findings from our study will contribute to the DDI information of SZA and SZB and, hopefully, facilitate future research on combining different components into one model.

It is beyond doubt that personalized medicine is the future direction of disease management. The integration of pharmacogenetics to design dosing regimens definitely leads to better pharmacotherapeutic outcomes; however, the pharmacogenomic information on Chinese traditional medicine preparations is sparse. Our study provides a practical strategy to evaluate the impact of CYP3A5 polymorphism on DDI caused by critical perpetrator compounds present in WZC. Elucidation of the influence of CYP3A5 polymorphism on tacrolimus metabolism allows more accurate DDI prediction using the PBPK modeling approach.

## 4. Materials and Methods

### 4.1. Chemicals and Reagents

Schizandrol A (purity ≥ 98%, lot: 58546–56-8) and Schizandrol B (purity ≥ 98%, lot: 58546–55-7) were purchased from PUSH Bio-Technology Co., Ltd. (Chengdu, China). CYP3cide (PF-4981517, lot: ECD192–1-PFZ), 6β-hydroxytestosterone (purity ≥ 98%, lot: A0277795) and nicotinamide adenine dinucleotide phosphate (NADPH, lot: LA50Q51) were obtained from J&K Technology Co., Ltd. (Beijing, China). Tacrolimus (purity ≥ 99.0%, lot: 050701130614) and testosterone (purity ≥ 99.0%, lot: L1415024) were obtained from Aladdin Biochemical Technology Co., Ltd. (Shanghai, China). Ascomycin (purity ≥ 99.0%, lot: D1202AS) and prednisolone (purity ≥ 98%, lot: J0402AS) were from Dalian Meilun Bio-Technology Co., Ltd. (Dalian, China). Pooled human-liver microsomes (HLMs, lot: 4133007) from 22 donors were obtained from BD Biosciences Co., Ltd. (New York, NY, USA). CYP3A5 *1/*3 HLMs (lot: 0710232) and CYP3A5 *3/*3 HLMs (lot: 0710253) were purchased from Transheep Co., Ltd. (Shanghai, China). All other reagents and solvents were from standard chemical suppliers and of analytical grade.

### 4.2. Analytical Instruments

All metabolic samples were analyzed by liquid chromatography–tandem mass spectrometry (LC-MS/MS). The details of bioanalytical protocol can be found in our previous publications [14,17] and Supplementary Data (Table S1).

### 4.3. CYP450 Enzyme-Inhibition Assay Protocols

#### 4.3.1. IC_50_ Shift Assay

IC_50_ shift assay was performed to discriminate reversible or irreversible inhibition caused by SZA and SZB. Pooled HLMs were used in this assay to determine the IC_50_ shift and identify time-dependent inhibition. The experiment was divided into two groups, depending on preincubation status. The basic incubation mixture with a total volume of 200 μL contained potassium phosphate buffer solution (PBS, 0.1 M, pH = 7.4), SZA (0, 20, 30, 40, 50, 60, 70, 80, 90, or 100 μM) or SZB (0, 0.0625, 0.125, 0.25, 0.5, 1, 2, 4, 8, 10, or 20 μM), MgCl_2_ (3 mM), NADPH (1 mM), testosterone (200 μM) and pooled HLMs (0.5 mg/mL). In all samples, the final concentrations of organic solvents were not more than 1% (V/V). Schizandrol (A or B) was pre-incubated with the pooled HLMs and testosterone for 3 min at 3 °C. In the groups without 30-minute preincubation, the reaction was started upon the addition of NADPH and terminated by 200 μL of ice-cooled methanol containing 0.315 μM of prednisolone (internal standard). For other groups, the reaction mixture with SZA/SZB and HLMs was pre-incubated with NADPH at 37 °C for 30 min. Next, testosterone was added to initiate the reaction. The reaction was terminated with the same method. All experiments were carried out in triplicates.

#### 4.3.2. Reversible Inhibition (RI) Assay

RI assay was used to measure the inhibitory constant (K_i_) of SZA or SZB on CYP3A (pooled HLMs), CYP3A4 (CYP3A5 * 3/*3 HLMs) and CYP3A5 (CYP3A5 * 1/*3 HLMs + CYP3cide) [37]. The basic incubation mixture with a total volume of 100 μL consisted of PBS (0.1 M, pH = 7.4), SZA (0, 4, 8, or 16 μM)/SZB (0, 1, 2, or 4 μM), NADPH (1 mM), tacrolimus at different concentrations and HLMs (0.2 mg/mL). The final concentration of organic solvents did not exceed 1% (*v*/*v*). For the K_i_ value of CYP3A, tacrolimus (0.25, 0.5, 1, or 2 μM) was added to the reaction mixture, followed by 3-minute pre-warming. The reactions started upon the addition of NADPH and were terminated after 10 min by adding 200 μL of ice-cooled acetonitrile containing 0.1 μM of ascomycin (internal standard). Similar operations were applied in the experiments with genotyped HLMs, except that a pre-warmed mixture of CYP3cide (1.2 μM) and CYP3A5 * 1/*3 HLM (0.2 mg/mL) was pre-incubated with NADPH for 10 min before adding the tacrolimus (0.5, 1, 2, or 4 μM) and SZA/SZB. The reaction was also terminated with ice-cooled acetonitrile. All experiments were carried out in triplicates.

#### 4.3.3. Time-Dependent Inhibition (TDI) Assay

A two-step protocol was followed to perform the general TDI assay. Firstly, SZA (0, 10, 16, 20, 25, or 32 μM)/SZB (0, 0.1, 0.2, 0.5, 1, or 2 μM) was incubated with pooled HLMs (0.5 mg/mL), PBS (0.1 M) and testosterone (200 μM) in a total volume of 200 μL, followed by 3-minute pre-warming at 37 °C in a shaking water bath. The reaction was initiated upon addition of NADPH (1 mM). At the designated time points (0, 5, 10, 20, or 30 min), an aliquot (20 μL) of the incubation mixture was transferred to a 180-microliter pre-warmed solution containing PBS (0.1 M), testosterone (200 μM) and NADPH (1 mM) for another 10-minute incubation. The reaction was stopped with ice-cooled acetonitrile containing prednisolone (0.315 μM). Similarly, the experiment for CYP3A4 followed the same steps described above, except for replacing pooled HLMs with CYP3A5 * 3/*3 HLMs with SZB at various concentrations (0, 0.5, 1, 2, 4, or 8 μM). For the CYP3A5 experiment, CYP3cide and CYP3A5 * 1/*3 HLMs were pre-warmed for 3 min before the reaction initiation. Various concentrations of SZA (0, 10, 20, 30, 40, or 50 μM) and SZB (0, 2, 4, 8, or 16 μM) were applied. All experiments were carried out in triplicates.

### 4.4. Data Analysis

#### 4.4.1. IC_50_ Values and Shift

IC_50_ values of SZA and SZB were determined by nonlinear regression analyses using GraphPad Prism version 5.0.0 (GraphPad Software, La Jolla, CA, USA). The formation of 6β-hydroxytestosterone in a control group was used as a surrogate marker for remaining enzymes’ activity. The formation rate was plotted against the logarithm concentration of the inhibitor (SZA or SZB). IC_50_ shift value was determined by Equation (2). A value of IC_50_ shift larger than 1.5 indicates time-dependent inhibition.

(2) $${\mathrm{IC}}_{50\;}\mathrm{shift}=\frac{{\mathrm{IC}}_{50}\;\mathrm{without}\;\mathrm{NADPH}\;\mathrm{preincubation}}{{\mathrm{IC}}_{50}\;\mathrm{with}\;\mathrm{NADPH}\;\mathrm{preincubation}}\;$$

#### 4.4.2. K_i_ Value

Dixon plots were used to determine the K_i_ value and the inhibitory type of SZA and SZB. Linear regression implemented in GraphPad Prism was plotted with the reciprocal value of metabolic reaction rate (1/v) against inhibitor concentration. The intersection of lines was used to calculate the K_i_ value.

#### 4.4.3. K_I_, k_inact_ and k_obs_ Values

The production rate of the 6β-hydroxytestosterone was used to demonstrate the remaining enzyme activity, which was plotted logarithmically against the preincubation time as abscissa. Kinetic parameters of the TDI (K_I_ and k_inact_ refer to the inhibitor concentration causing half-maximal inactivation and the maximal inactivation rate constant, respectively) were determined with Equation (3). k_obs_ refers to the observed inactivation rate of the affected enzyme, determined by the negative slope of the linear regression. k_inact_ is calculated with the negative reciprocal value of the straight line at the Y-intercept, while the interception on *X*-axis represents K_I_. [I] stands for the inhibitor concentration. A high ratio of k_inact_ over K_I_ indicates great inhibitory potency.

(3) $$k_{\mathrm{obs}}=\frac{k_{\mathrm{inact}}\times \left[I\right]}{K_{I}+\left[I\right]}\;$$

### 4.5. Model Development

Parameters of the PBPK model are listed in Table 3. All physicochemical and in vitro pharmacokinetic parameters were obtained from the literature or predicted by Simcyp^®^ Simulator (version 16, Certara, Sheffield, U.K.). The intrinsic clearance value of pooled HLMs and human tacrolimus PBPK model was retrieved from our previous publication [14]. The models for CYP3A5 expressers/non-expressers were built by modifying our established model with intrinsic clearance values of each CYP isoform [17,19]. With the inhibitory parameters acquired from our experiments, a DDI module was incorporated into the PBPK model of tacrolimus.

## 5. Conclusions

In the present study, the inhibitory effects of SZA/SZB on CYP3 A4 and CYP3A5 were identified. Subsequently, the acquired information was successfully integrated into a PBPK model to quantify the DDI between the SZA/SZB and the tacrolimus, which was further complicated by the CYP3A5 genotype. The information obtained in this study improves our understanding of the DDI between WZC and tacrolimus. Clearly, PBPK modeling has emerged as a powerful approach to examine herb-involved DDI; special attention should be paid to the combined use of WZC and tacrolimus in clinical practice.

## Figures and Tables

**Table 1:** Predicted and observed values for pharmacokinetic parameters of SZA and SZB.

PK Parameters	SZA			SZB		
	Pre ^1^	Obs ^2^	FE ^3^	Pre	Obs	FE
C_max_^4^ (ng/mL)	126.88	111.37	1.14	66.70	65.18	1.02
T_max_^5^ (h)	0.96	1.81	1.90	0.73	1.13	1.54
AUC(ng/mL·h)	437.80	467.14	1.10	271.74	242.97	1.12

^1^ Pre: predicted; ^2^ Obs: observed; ^3^ FE: fold error; ^4^ C_max_: total maximal concentration in plasma; ^5^ T_max_: the time after administration at which the plasma drug concentration reaches C_max_.

**Table 2:** Alterations in tacrolimus exposure after a single dose or multiple doses of SZA and SZB in CYP3A5 expressers and non-expressers.

Population	Inhibitors	Dose Regimen	RI Case#1	TDI Case#2	RI and TDI Case#3
CYP3A5 Non-expresser	SZA	Single dose	—	118.07 ^1^/118.17 ^2^ 1.00 ^3^	—
		Multiple doses	—	118.07/136.76 1.16	—
	SZB	Single dose	118.07/128.33 1.09	118.07/124.60 1.06	118.07/135.01 1.14
		Multiple doses	118.07/148.65 1.26	118.07/173.14 1.47	118.07/185.61 1.57
CYP3A5 Expresser	SZA	Single dose	—	62.50/62.54 1.00	—
		Multiple doses	—	62.50/62.66 1.00	—
	SZB	Single dose	62.50/69.83 1.12	62.50/64.72 1.04	62.50/72.12 1.15
		Multiple doses	62.50/70.68 1.13	62.50/70.60 1.13	62.50/76.68 1.23

^1^ The predicted AUC of tacrolimus alone; ^2^ the predicted AUC of tacrolimus after coadministration with inhibitor (unit, ng/mL·h); ^3^ AUC ratio (AUCR) between coadministration with inhibitor and without inhibitor.

**Table 3:** Input parameters of PBPK simulations.

Parameters	SZA	SZB	
	Value	Value	Reference
Molecular weight (g/mol)	432.52	416.47	-
Ionization pattern	Neutral	Neutral	-
Log P_o:w_^1^	3.390	3.380	Predicted by ADMET Predictor ^11^
f_u, plasma_^2^	0.083	0.084	Predicted by Simcyp^®^
B/P ^3^	1.254	0.785	Predicted by Simcyp^®^
**Absorption phase**			
Model	ADAM -		
f_a_^4^	0.500	0.734	Optimized using Simcyp^®^
k_a_ (1/h) ^5^	0.218	0.437	Optimized using Simcyp^®^
P_eff, human_ (×10^−4^ cm/s) ^6^	0.500	1.000	Optimized using Simcyp^®^
**Distribution phase**			
Model	Full PBPK		
V_ss_ (L/kg) ^8^	2.516	2.225	Method 1 ^12^
K_p_ scalar ^9^	0.5	1	Optimized using Simcyp^®^
**Elimination phase**			
Model	Whole-Organ Metabolic Clearance		
CL_int,HLM_ (μL/min/mg) ^10^ protein) ^7^	50	4.5	In-house data

^1^ Log P_o:w_: the partition coefficient in oil and water; ^2^ f_u__,plasma_: unbound fraction in plasma; ^3^ B/P: blood to plasma ratio; ^4^ f_a_: the fraction of drug dose entering the cellular space of the enterocytes; ^5^ k_a_: the first-order absorption rate constant; ^6^ P_eff, human_: the effective intestinal permeability in humans; ^7^ PSA: polar surface area; ^8^ V_ss_: the volume of distribution; ^9^ K_p_ scalar: scaling factor for tissue to plasma partition coefficient; ^10^ CL_int, HLM_: intrinsic clearance in human liver microsomes; ^11^ ADMET Predictor Module in GastroPlus™ (version 9.0, Simulations Plus, Inc., Lancaster, CA, USA) was used for prediction. ^12^ Method 1: based on the approach of Poulin and Theil [38] with the correction by Berezhkovskiy [39].

## Data Availability

Data are contained within the article or Supplementary Material.

## Supplementary Materials

The following are available online at https://www.mdpi.com/article/10.3390/ijms23094485/s1.

## Abbreviations

Absorption, distribution, metabolism and excretion	ADME
Area under the curve AUC ratio	AUC AUCR
Clinical Pharmacogenetics Implementation Consortium	CPIC
Cytochrome P450	CYP450
Drug–drug interaction	DDI
Fold error	FE
High-performance liquid chromatography	HPLC
Human-liver microsome 50% inhibitory concentration	HLM IC_50_
Reversible inhibition constant	K_i_
Observed inactivation rate of the affected enzyme	k_obs_
Maximum inactivation rate constant	k_inact_
Inhibitor concentration causing half-maximal inactivation	K_I_
Mass spectrometry	MS
Nicotinamide adenine dinucleotide phosphate	NADPH
Physiologically based pharmacokinetic	PBPK
Area under the curve ratio	AUCR
Reversible inhibition	RI
Schisandrin A	SIA
Schisantherin A	STA
Schizandrol A	SZA
Schizandrol B	SZB
Tacrolimus	FK-506
Time-dependent inhibition	TDI
Traditional Chinese medicine	TCM
Wuzhi capsule	WZC
