# Supplementary material for: Examination of the Impact of CYP3A4/5 on Drug–Drug Interaction between Schizandrol A/Schizandrol B and Tacrolimus (FK-506): A Physiologically Based Pharmacokinetic Modeling Approach
Source: Int J Mol Sci. 2022 Apr 19;23(9):4485. doi: 10.3390/ijms23094485 (PMC9103789; doi:10.3390/ijms23094485)
Supplement: Supplementary file 1 [file ijms-23-04485-s001.zip › ijms-1663551-supplementary.pdf]

Examination of the Impact of CYP3A4/5 on Drug–Drug Interaction between Schizandrol A/Schizandrol B and Tacrolimus (FK-506): A Physiologically Based Pharmacokinetic Modeling Approach

Qingfeng He [1,†], Fengjiao Bu [1,†], Qizhen Wang [1], Min Li [1], Jiaying Lin [1], Zhijia Tang [1], Wen Yao Mak [1,3], Xiaomei Zhuang [4], Xiao Zhu [1], Hai-Shu Lin [2,*] and Xiaoqiang Xiang [1,*]

1 Department of Clinical Pharmacy and Pharmacy Administration, School of Pharmacy, Fudan University, Shanghai 201203, China; qf_he@fudan.edu.cn (Q.H.); fbu13@fudan.edu.cn (F.B.); wangqz16@fudan.edu.cn (Q.W.); 19211030067@fudan.edu.cn (M.L.); 21211030054@fudan.edu.cn (J.L.); zjtang@fudan.edu.cn (Z.T.); xiaozhu@fudan.edu.cn (X.Z.)
2 College of Pharmacy, Shenzhen Technology University, Shenzhen 518118, China
3 Clinical Research Centre, Institute for Clinical Research, National Institute of Health, Hospital Pulau Pinang, Pinang 10450, Malaysia; makwenyao@gmail.com
4 State Key Laboratory of Toxicology and Medical Countermeasures, Beijing Institute of Pharmacology and Toxicology, Beijing 100850, China; xiaomeizhuang@163.com
* Correspondence: linhaishu@sztu.edu.cn (H.-S.L.); xiangxq@fudan.edu.cn (X.X.); Tel.: +86-21-51980024 (X.X.)
† Authors contributed equally to this manuscript.

Table S1. Summary of LC-MS/MS parameters

	Tacrolimus	Ascomycin (IS)	6β-Hydroxytestosterone	Prednisolone (IS)
**MRM Transition m/z (Q1-Q3)**	821.5>768.6	305.2>269.2	810.7>757.6	361.3>343.2
**MS Ionization**	Electrospray ionization (ESI) mode			

**DP (V)**	25.0	78.0	100.0	22.0
**CE (V)**	30.0	22.0	30.0	15.0
**Column**	Hypersil Gold reversed-phase C18 column (2.1 × 100 mm, 3 μm) protected by a 12.5-mm Agilent Eclipse XDB-C18 guard column			
**Column Temperature**	35ºC			
**Run Time**	4 min			
**Mobile Phase**	Phase A: acetonitrile<br>Phase B: 10 mM ammonium acetate (containing 0.1% acetic acid) with isocratic elution of A:B = 95:5		Phase A: acetonitrile<br>Phase B: 0.1% formic acid (A:B = 60:40)	
**Flow**	0.3 mL/min			
**Injection volume**	5 μL			
**Retention Time**	3.75 min	2.79 min	3.75 min	2.79 min
